# Harnessing the drucebo effect: A new frontier in podiatric patient care

**DOI:** 10.1002/jfa2.70029

**Published:** 2025-01-12

**Authors:** Roberto Tedeschi

**Affiliations:** ^1^ Department of Biomedical and Neuromotor Sciences Alma Mater Studiorum University of Bologna Bologna Italy

Dear Editor,

The drucebo effect, a recently conceptualized phenomenon, highlights the intricate interplay between patient expectations, therapeutic outcomes, and the clinician–patient relationship. Defined as the blend of drug or treatment effects and placebo/nocebo phenomena, this effect underscores the potential of leveraging patient psychology to enhance therapeutic efficacy. In podiatry, where treatment modalities often combine pharmacological, physical, and surgical interventions, integrating an understanding of the drucebo effect into clinical practice may yield transformative benefits [1, 2].

Recent studies have elucidated the significant role of patient expectations in modulating therapeutic outcomes, particularly through mechanisms of anticipatory neurochemical changes and altered pain perception. Although the application of these findings has gained traction in pharmacology and physiotherapy, podiatry lags behind in adopting this evidence‐based approach. Yet, the unique nature of podiatric care—emphasizing physical interventions, biomechanical corrections, and patient education—positions the field as an ideal candidate for implementing drucebo‐focused strategies [3, 4].

The integration of the drucebo effect into podiatric practice entails addressing several key dimensions:

1. **Patient Expectations and Communication:** Empirical evidence highlights that patient expectations, shaped through transparent and empathetic clinician communication, directly influence treatment adherence, perceived pain, and functional outcomes. For instance, structured patient education about realistic outcomes can mitigate anxiety and enhance satisfaction, particularly in chronic pain syndromes such as plantar fasciitis or hallux valgus.

**Therapeutic Environment:** Optimizing the therapeutic milieu by fostering trust and setting achievable goals can amplify the positive impacts of interventions. The psychological burden associated with foot and ankle disorders, often linked to reduced mobility and independence, necessitates a holistic approach that integrates psychological support alongside clinical treatments.

**Empirical Research:** Despite its promise, the drucebo effect remains underexplored in podiatric medicine. Future studies should investigate its clinical relevance through randomized controlled trials, focusing on conditions with substantial psychosomatic components. Additionally, the development of guidelines to standardize communication strategies and expectation management in podiatric settings is crucial.

Incorporating the drucebo effect aligns with the broader paradigm shift toward patient‐centered care, which recognizes the integral role of psychological factors in recovery. It is worth noting that podiatric disorders often have complex biopsychosocial dimensions, further emphasizing the relevance of this approach. Beyond individual patient outcomes, leveraging the drucebo effect could contribute to improved healthcare efficiency by reducing treatment‐related nonadherence and enhancing the cost‐effectiveness of interventions [5].

This correspondence aims to encourage a re‐evaluation of current practices in podiatric medicine, advocating for a systematic incorporation of drucebo principles. Such an approach not only has the potential to enhance patient outcomes but also underscores the importance of integrating psychological insights into evidence‐based podiatry.

## AUTHOR CONTRIBUTIONS

The author has accepted responsibility for the entire content of this manuscript and approved its submission.

## FUNDING INFORMATION

The author states that there is no funding involved.

## CONFLICT OF INTEREST STATEMENT

The author declares no conflicts of interest.

## ETHICAL APPROVAL

Not applicable.

## CONSENT

Not applicable.

## REGISTRATION OF RESEARCH STUDIES

Not applicable.

## Data Availability

Research data are not shared.
